# Supplementary material for: What are the general public’s expectations about the likely duration of common acute infections? A cross-sectional survey of Australian residents
Source: BMJ Open. 2024 Dec 22;14(12):e090190. doi: 10.1136/bmjopen-2024-090190 (PMC11667410; doi:10.1136/bmjopen-2024-090190)
Supplement: online supplemental material 1 [file bmjopen-14-12-s001.docx]

**Supplementary material 1 Dynata Australia Demographic***


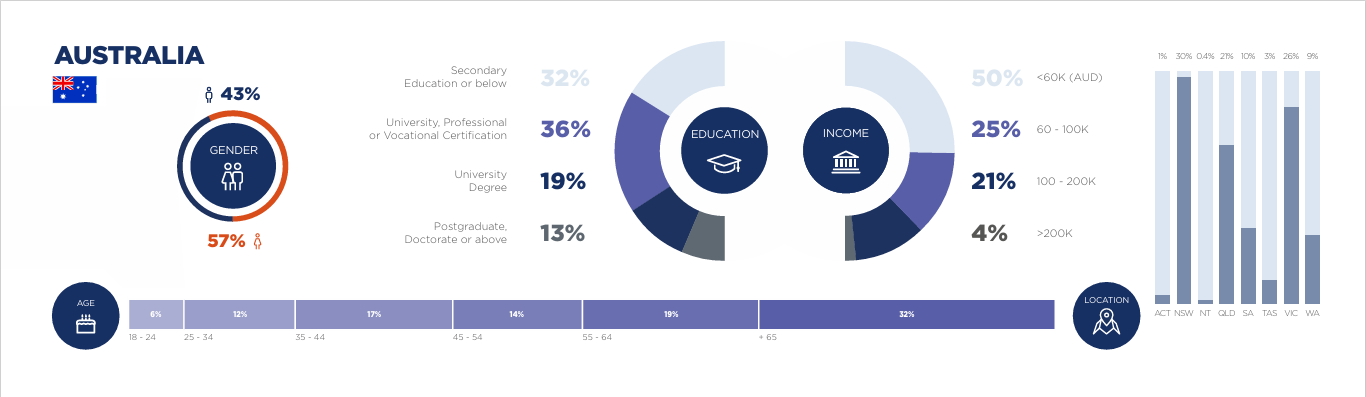


* The screenshot comes from the informational documents that Dynata provided to the researchers at the start of the study, with permission to share.

**Supplementary material 2 Survey questions**

*Dear participants, researchers at the Institute for Evidence-based Healthcare at Bond University are conducting a study about common infections (e.g., sore throat, common cold).*

*We wish to explore how long people think that a number of these infections typically last and what influences peoples' decisions to seek care for these infections.*

*The study findings will help design tailored educational and targeted public health strategies to improve publics' understanding of the duration of common infections and support people to make informed choices for managing these infections.*

*The survey will take an average of 25-30 minutes to complete.*

**People's expectations about the natural history of common acute infections: an Australia-wide survey**

***Section A***

| 1. **Which age group (years) applies to you?** | | | | |  |  |
| --- | --- | --- | --- | --- | --- | --- |
| 🞎 ≤25 | 🞎 26-35 | 🞎 36-45 | 🞎 46-55 | 🞎 ≥56 | |  |
| 1. **What is your gender?** | | | | |  |  |
| 🞎 Male | 🞎 Female | 🞎 Other |  |  | |  |
| 1. **What is your level of education? Please indicate the highest level of education you have completed.** | | | | |  |  |
| 🞎 High school certificate | | |  |  | |  |
| 🞎 Diploma, Certificate I-IV, or apprenticeship | | | |  | |  |
| 🞎 Undergraduate degree: Bachelor's degree or equivalent | | | |  | |  |
| 🞎 Postgraduate degree: Masters or Doctoral degree | | | | | |  |
| 1. **Is English the main language you speak at home?** | | | | |  |  |
| 🞎 Yes | 🞎 No |  |  |  | |  |
|  | |  |  |  |  | |
| 1. **Do you identify as an Aboriginal and/or Torres Strait Islander?** | | | | |  |  |
| 🞎 Yes | 🞎 No |  |  |  | |  |
| 1. **Please enter your postcode:** | | | | |  |  |
| 1. **What is your current household situation?** | | | | |  |  |
| 🞎 Single | |  |  |  | |  |
| 🞎 Single with children | |  |  |  | |  |
| 🞎 Married, living with a partner | |  |  |  | |  |
| 🞎 Married, living with a partner and children | | | |  | |  |
| 🞎 Other | |  |  |  | |  |
|  | | |  |  |  |  |
| 1. **Do you have children younger than 18 years of age living at home?** | | | | |  |  |
| 🞎 Yes | 🞎 No |  |  |  | |  |
|  |  |  |  |  | |  |
| 8b**. If yes to the previous question, how many?** | | | | |  |  |
| 🞎 1 | 🞎 2 | 🞎 3 | 🞎 4 or more |  | |  |
| 1. **What is your current employment status?** | | | | |  |  |
| 🞎 Full time (more than 38 hours per week) | | |  | | |  |
| 🞎 Part-time | | |  | | |  |
| 🞎 Casual | | |  | | |  |
| 🞎 Not currently in paid employment | | |  | | |  |
| 1. **Annual gross household income ($)** | | | | |  |  |
| 🞎 <40,000 | | | |  | |  |
| 🞎 40,001-60,000 | | | |  | |  |
| 🞎 60,0001-80,000 | | | |  | |  |
| 🞎 80,001-100,000 | | | |  | |  |
| 🞎 >100,001 | | | |  | |  |
| 🞎 Prefer not to say.   1. Do you have any of the following chronic medical conditions? | | | |  | |  |
| 🞎 Chronic Obstructive Pulmonary Disease (COPD) | | | | | | |
| 🞎 Asthma | | | | | | |
| 🞎 Recurrent urinary tract infections | | | | | | |
| 🞎 No  🞎 Prefer not to say. | | | | | | |
| 1. **Are you a Medicare cardholder?** | | | | |  |  |
| 🞎 Yes | 🞎 No |  |  |  | |  |
|  |  |  |  |  | |  |
| 1. **The next question is about your preferences, generally, when it comes to how much health care you prefer to receive.**   Sometimes, medical action is clearly necessary, and sometimes it is clearly not necessary. People often differ in their beliefs about whether medical action is needed.  **In situations where it is not clear, do you tend to lean towards taking action or do you lean towards waiting and seeing if action is needed?** | | | | | |  |
| Importantly, please note there is no "correct" answer.  Please answer on the scale of 1-6 below   \| I strongly lean toward waiting and seeing. \| I lean toward waiting and seeing. \| I somewhat lean toward waiting and seeing. \| I somewhat lean toward taking action. \| I lean toward taking action. \| I strongly lean toward taking action. \| \| --- \| --- \| --- \| --- \| --- \| --- \| \| 1  🞎 \| 2  🞎 \| 3  🞎 \| 4  🞎 \| 5  🞎 \| 6  🞎 \| | | | | |  |  |
|  | | | | |  |  |

***Section B***

The next set of questions are about your decision to seek medical care for common infections (acute self-limiting infections), like cough and cold, and issues that might influence that decision.

**"Acute infections"** are infections that are of quick onset and usually of short duration.

"**Self-limiting infections**" mean infections that usually get better by themselves without needing treatment (other than managing symptoms such as pain or fever with appropriate medications)

For all questions, please assume that the symptoms mentioned are **NOT related to COVID-19.**

1. If you develop symptoms of a self-limiting acute infection (as defined previously) such as cough, common cold, or sore throat, in general how likely are you to see a General Practitioner about it? Select from the options below.

|  |  |  |  |  |  |
| --- | --- | --- | --- | --- | --- |
| 🞎 Extremely likely | | | | | |
| 🞎 Likely | | | | | |
| 🞎 Neither likely nor unlikely | | | | | |
| 🞎 Unlikely | | | | | |
| 🞎 Extremely unlikely | | | | | |
|  | | | | | |

1. If you decide to see your GP, what factors might influence your decision? …………………..
2. If it was your CHILD who was experiencing symptoms of acute infections (e.g., cough, common cold, sore throat), what factors might influence your decision to see a GP? ……………………………

**Section C**

For the next set of questions, please estimate how many days you think each of the infections listed typically last for and what might make you to decide to seek healthcare for the illness.

Please assume the symptoms are **NOT related to COVID-19**.

| **COUGH** |
| --- |
| A1. Imagine that you developed a COUGH. How long (that is, how many **days**) do you typically expect it to last? (i.e., from when the cough starts until it is gone)?  Please specify…………………………… |
| **A2**. After about how many **days** of having a COUGH might you decide to see a GP about it?  Please specify (If you are unlikely to *visit a GP about a COUGH, enter '0') ………………….* |
| A3. In most people, **COUGH** gets better within about 14-21 days, without any treatment other than managing symptoms.  Do you have any concerns waiting for the **COUGH** to get better on its own and not seeking healthcare (beyond using over-the-counter medications or home remedies)?  Please describe your concerns…………………………………… |
| A4. Which of the following possible reasons for seeking healthcare for a COUGH are the most important to you  [Please rank these in order of importance]. Rank all 6 boxes (including 'other') to proceed to the next question. If you don’t have any other reasons to suggest, please rank “other” as the 6^th^ reason and write ‘n/a’ in the text box. |
| 🞎 Wanting to prevent possible complications.  🞎 Wanting to get better faster |
| 🞎 Not getting better with over-the-counter medicines or home remedies |
| 🞎 Symptoms taking too long to get better.  🞎 Wanting to reduce illness impact on daily life/ sleep. |
| 🞎 Other, please specify…………………………… |

| **COMMON COLD** |
| --- |
| B1. Imagine that you developed a COMMON COLD. How long (that is, how many **days**) do you typically expect it to last? (i.e., from when the cold starts until it is gone)?  Please specify…………………………… |
| B2. After about how many **days** of having a COMMON COLD might you decide to see a GP about it?  Please specify (If you are unlikely to *visit a GP about a COMMON COLD, enter ‘0’) ………………………* |
| B3. In most people, **COMMON COLD** gets better within about 3-10 days without any treatment other than for managing symptoms.    Do you have any concerns waiting for a **COMMON COLD** to get better on its own and not seeking health care (beyond using over-the-counter medications such as ‘cold and flu’ tablets containing decongestants and paracetamol, or home remedies)? Please describe your concerns………………… |
| B4. Which of the following reasons for seeking healthcare for a COMMON COLD are the most important to you?  [Please rank these in order of importance] Rank all 6 boxes (including ‘other’) to proceed to the next question. If you don’t have any other reasons to suggest, please rank “other” as the 6^th^ reason and write ‘n/a’ in the text box. |
| 🞎 Wanting to prevent possible complications |
| 🞎 Not getting better with over-the-counter medicines or home remedies |
| 🞎 Symptoms taking too long to get better.  🞎 Wanting to reduce illness impact on daily life/ sleep.  🞎 Wanting to get better faster |
| 🞎 Other, please specify…………………………… |

| **SORE THROAT** |
| --- |
| C1. Imagine that you developed a SORE THROAT. How long (that is, how many **days**) do you typically expect it to last? (i.e., from when the sore throat starts until it is gone)?  Please specify…………………………………. |
| C2. After how many **days** of having a SORE THROAT might you decide to see a GP about it?  Please specify (If you are unlikely to *visit a GP about a SORE THROAT, enter ‘0’) ………………………….* |
| C3. In most people, **SORE THROAT** gets better within about 2-7 days, without any treatment other than for managing symptoms.  Do you have any concerns waiting for a **SORE THROAT** to get better on its own and not seeking healthcare (beyond using over-the-counter medications such as paracetamol for pain and fever or home remedies)? Please describe your concerns…………………. |
| C4. Which of the following reasons for seeking healthcare for a SORE THROAT are the most important to you?  [Please rank these in order of importance] Rank all 6 boxes (including ‘other’) to proceed to the next question. If you don’t have any other reasons to suggest, please rank “other” as the 6^th^ reason and write ‘n/a’ in the text box. |
| 🞎 Wanting to prevent possible complications |
| 🞎 Not getting better with over-the-counter medicines or home remedies |
| 🞎 Symptoms taking too long to get better.  🞎 Wanting to reduce illness’s impact on daily life/ sleep.  🞎 Wanting to get better faster |
| 🞎 Other, please specify…………………………… |

| **MIDDLE EAR INFECTION (EAR PAIN)** |
| --- |
| D1. Imagine that you developed a MIDDLE EAR INFECTION. How long (that is, how many **days**) do you typically expect it to last? (i.e., from when the MIDDLE EAR INFECTION starts until it is gone)?  Please specify……………………………………... |
| D2. After how many **days** of having a MIDDLE EAR INFECTION, might you decide to see a GP about it?  Please specify (If you are unlikely to *visit a GP about a MIDDLE EAR INFECTION, enter ‘0’) ………………………...* |
| D3. In most people, a **MIDDLE EAR INFECTION** gets better within about 3-7 days, without any treatment other than for managing symptoms.  Do you have any concerns about waiting for a **MIDDLE EAR INFECTION** to get better on its own and not seeking healthcare (beyond using over-the-counter medications such as paracetamol for pain and fever, or home remedies)?  Please describe your concerns ……………………………………. |
| D4. Which of the following reasons for seeking healthcare for a MIDDLE EAR INFECTION are the most important to you?  [Please rank these in order of importance] Rank all 6 boxes (including ‘other’) to proceed to the next question. If you don’t have any other reasons to suggest, please rank “other” as the 6^th^ reason and write ‘n/a’ in the text box. |
| 🞎 Wanting to prevent possible complications |
| 🞎 Not getting better with over-the-counter medicines or home remedies |
| 🞎 Symptoms taking too long to get better.  🞎 Wanting to reduce illness’s impact on daily life/ sleep.  🞎 Wanting to get better faster |
| 🞎 Other, please specify…………………………… |
| **SINUSITIS (SINUS PAIN)** |
| E1. Imagine that you developed SINUS PAIN. How long (that is, how many **days**) do you typically expect it to last? (i.e., from when the SINUS PAIN starts until it is gone)?  Please specify………………………………... |
| E2. After how many **days** of having a SINUS PAIN might you decide to see a GP about it?  Please specify (If you are unlikely to *visit a GP about SINUSITIS, enter ‘0’) ………………………...* |
| E3. In most people, a **SINUS PAIN** gets better within about 14 days, without any treatment other than for managing symptoms.  Do you have any concerns waiting for the **SINUS PAIN** to get better on its own and not seeking healthcare (beyond using over-the-counter medications, such as saline or steroid spray, or home remedies)?  Please describe your concerns…………………………………. |
| E4. Which of the following reasons for seeking healthcare for SINUS PAIN are the most important to you?  [Please rank these in order of importance]. Rank all 6 boxes (including ‘other’) to proceed to the next question. If you don’t have any other reasons to suggest, please rank “other” as the 6^th^ reason and write ‘n/a’ in the text box.   \| 🞎 Wanting to prevent possible complications \| \| --- \| \| 🞎 Not getting better with over-the-counter medicines or home remedies \| \| 🞎 Symptoms taking too long to get better.  🞎 Wanting to reduce illness’s impact on daily life/ sleep.  🞎 Wanting to get better faster \| \| 🞎 Other, please specify…………………………… \| |

| **CONJUNCTIVITIS (PINK EYE)** |
| --- |
| F1. Imagine that you developed symptoms of CONJUNCTIVITIS. How long (that is, how many **days**) do you typically expect it to last? (i.e., from when the CONJUNCTIVITIS starts until it is gone)?  Please specify…………………………………………... |
| F2. After how many **days** of having CONJUNCTIVITIS might you decide to see a GP about it?  Please specify (If you are unlikely to *visit a GP about a CONJUNCTIVITIS, enter ‘0’) …………………………* |
| F3. In most people, **CONJUNCTIVITIS** gets better within about 7 days, without any treatment other than for managing symptoms.  Do you have any concerns about waiting for **CONJUNCTIVITIS** to get better on its own and not seeking healthcare (beyond using over-the-counter medications, such as anti-allergy eye drops, or home remedies)?  Please describe your concerns……………………... |
| F4. Which of the following possible reasons for seeking healthcare for conjunctivitis are the most important to you?  [Please rank these in other of importance] Rank all 6 boxes (including ‘other’) to proceed to the next question. If you don’t have any other reasons to suggest, please rank “other” as the 6^th^ reason and write ‘n/a’ in the text box. |
| 🞎 Wanting to prevent possible complications |
| 🞎 Not getting better with over-the-counter medicines or home remedies |
| 🞎 Symptoms taking too long to get better.  🞎 Wanting to reduce illness’s impact on daily life/ sleep.  🞎 Wanting to get better faster |
| 🞎 Other, please specify…………………………… |

UTI QUESTION – to be asked of females only.

| **UNCOMPLICATED URINARY TRACT INFECTIONS (UTI) [symptoms associated with pain or burning feeling when urinating, passing frequent or small amount of urine]** |
| --- |
| G1. Imagine that you developed symptoms of **URINARY TRACT INFECTION**. How long (that is, how many **days**) do you typically expect it to last? (i.e., from when the Urinary Tract Infection starts until it is gone)?  Please specify……………………………………... |
| G2. After how many **days** of having symptoms of **URINARY TRACT INFECTION** might you decide to see a GP about it?  Please specify (If you are unlikely to *visit a GP about a URINARY TRACT INFECTION, enter ‘0’) ……………………………* |
| G3. In many women, a **URINARY TRACT INFECTION** gets better on its own by about 9 days, without any treatment other than managing symptoms.  Do you have any concerns waiting for the symptoms of **URINARY TRACT INFECTION** to get better on its own and not seeking healthcare (beyond using over-the-counter medications, such as ibuprofen for pain management or fever, or home remedies)?  Please describe your concerns…………………………………………. |
| G4. Which of the following possible reasons for seeking healthcare for a urinary tract infection are the most important to you?  [Please rank these in order of importance] Rank all 6 boxes (including ‘other’) to proceed to the next question. If you don’t have any other reasons to suggest, please rank “other” as the 6^th^ reason and write ‘n/a’ in the text box. |
| 🞎 Wanting to prevent possible complications |
| 🞎 Not getting better with over-the-counter medicines or home remedies |
| 🞎 Symptoms taking too long to get better.  🞎 Wanting to reduce illness’s impact on daily life/ sleep.  🞎 Wanting to get better faster |
| 🞎 Other, please specify…………………………… |

| **UNCOMPLICATED IMPETIGO (SCHOOL SORES)** |
| --- |
| H0: Have you heard of Impetigo (also known as School sores)? Only proceed to showing H1-5 if participants answer Yes.  Yes [ ] No [ ] |
| H1. Imagine that you developed symptoms of SCHOOL SORES. How long (that is, how many **days**) do you typically expect it to last? (i.e., from when the school sore starts until it is gone)?  Please specify………………... |
| H2. After how many **days** of having SCHOOL SORES would you decide to see a GP about it?  Please specify (If you are unlikely to *visit a GP about SCHOOL SORES, enter ‘0’)* ………………………………. |
| H3. In most people, SCHOOL SORES get better on their own by about 7-14 days, without any treatment other than for managing symptoms.  Do you have any concerns waiting for SCHOOL SORES to get better on its own and not seeking healthcare (beyond using over-the-counter medications, such as povidone iodine shampoo, or home remedies)?  Please describe your concerns……………………………… |
| H4. Which of the following possible reasons for seeking healthcare for SCHOOL SORES are the most important to you?  [Please rank these in order of importance] Rank all 6 boxes (including ‘other’) to proceed to the next question. If you don’t have any other reasons to suggest, please rank “other” as the 6^th^ reason and write ‘n/a’ in the text box. |
| 🞎 Wanting to prevent possible complications |
| 🞎 Not getting better with over-the-counter medicines or home remedies |
| 🞎 Symptoms taking too long to get better.  🞎 Wanting to reduce illness’s impact on daily life/ sleep.  🞎 Wanting to get better faster |
| 🞎 Other, please specify…………………………… |

**Supplementary material 3 Participant flow chart**

Potential participants who responded to the survey

(n=1288)

**Excluded (n=699)**

1. Participants who failed ReCAPTCHA test (n=51)

2. Participants who spent < 8 minutes answering the questions (n=372)

3. Participants who failed other quality scores check; straight liners including those who consistently selected the same answers across multiple questions (n=228)

4. Disqualified: bad open-ends, profanity (n=48)

Number of responses included for analysis.

(n=589)

**Supplementary material 4** Participant characteristics (n=589, unless stated otherwise)

| **Characteristic** | **n (%)** |
| --- | --- |
| **Age category (years)** |  |
| ≤25 | 27 (4.6) |
| 26-35 | 101 (17.2) |
| 36-45 | 110 (18.7) |
| 46-55 | 104 (17.7) |
| ≥56 | 247 (41.9) |
| **Gender** |  |
| Male | 239 (40.6) |
| Female | 348 (59.1) |
| Other | 2 (0.3) |
| **Education** |  |
| High school certificate | 167 (28.4) |
| Diploma or Certificate I-IV, or Apprenticeship | 191 (32.4) |
| Undergraduate degree: Bachelor's degree | 155 (26.3) |
| Postgraduate degree: Master's or Doctor | 76 (12.9) |
| **English spoken as the main language at home** |  |
| Yes | 550 (93.4) |
| **Aboriginal and/or Torres Strait Islander** |  |
| Yes | 21 (3.6) |
| **State or Territory in Australia** |  |
| New South Wales | 183 (31.1) |
| Victoria | 146 (24.8) |
| Queensland | 125 (21.2) |
| South Australia | 52 (8.8) |
| Western Australia | 48 (8.2)) |
| Australian Capital Territory | 16 (2.7) |
| Tasmania | 16 (2.7) |
| Northen Territory | 3 (0.5) |
| **Australian Region** |  |
| Metro | 401 (68.1) |
| Regional Area | 188 (31.9) |
| **Current household situation** |  |
| Single | 170 (28.9) |
| Single with children | 49 (8.3) |
| Married, living with a partner | 200 (34.0) |
| Married, living with a partner and children | 140 (23.8) |
| Other | 30 (5.1) |
| **Living with children ≤18 years** |  |
| Yes | 170 (28.9) |
| **If living with children, how many? (n=170) ^a^** |  |
| 1 | 70 (41.2) |
| 2 | 68 (40) |
| 3 | 20 (11.8) |
| ≥ 4 | 12 (7.1) |
| **Employment status** |  |
| Full time (≥ 38 hours/ week) | 193 (32.8) |
| Part-time | 106 (18) |
| Casual | 28 (4.8) |
| Not currently in paid employment | 262 (44.5) |
| **Annual household income (AUD $)** |  |
| <40,000 | 126 (21.4) |
| 40,001-60,000 | 103 (17.5) |
| 60,0001-80,000 | 81 (13.8) |
| 80,001-100,000 | 72 (12.2) |
| >100,001 | 173 (29.4) |
| Prefer not to say | 34 (5.8) |
| **Medicare holder** |  |
| Yes | 564 (95.8) |
| **Have this pre-existing medical condition?** |  |
| Chronic Obstructive Pulmonary Diseases | 22 (3.7) |
| Asthma | 92 (15.6) |
| Recurrent Urinary Tract Infections | 27 (4.6) |
| **Maximizer-Minimizer Scale ^b^** |  |
| Maximizer | 243 (41.3) |
| Minimizer | 346 (58.7) |
| **Health Seeking for acute self-limiting infections ^c^** |  |
| Likely | 138 (23.4) |
| Neither likely nor unlikely | 85 (14.4) |
| Unlikely | 366 (62.1) |

^a^ The denominator here is the number of participants who answered “Yes” to living with children ≤18 years (n= 170)

^b^ MMI (Maximizer-Minimizer scale) assesses patients’ preference for aggressive versus a more passive approach to healthcare.

^c^ This question explored participants’ likelihood of visiting the GPs with acute self-limiting infection.
