# Supplementary material for: What are the general public’s expectations about the likely duration of common acute infections? A cross-sectional survey of Australian residents
Source: BMJ Open. 2024 Dec 22;14(12):e090190. doi: 10.1136/bmjopen-2024-090190 (PMC11667410; doi:10.1136/bmjopen-2024-090190)
Supplement: Supplementary file 2 [file bmjopen-2024-090190.reviewer_comments.pdf]

PEER REVIEW HISTORY

BMJ Open publishes all reviews undertaken for accepted manuscripts. Reviewers are asked to complete a checklist review form and are provided with free text boxes to elaborate on their assessment. These free text comments are reproduced below.

ARTICLE DETAILS

Title (Provisional)

What are peoples' expectations about the likely duration of common acute infections? A cross-sectional survey of the Australian public.

Authors

Boaitey, Kwame Peprah; Bakhit, Mina; Jones, Mark; Hoffmann, Tammy

---

VERSION 1 - REVIEW

**Reviewer**	**1**
**Name**	**Kurotschka, Peter Konstantin**
**Affiliation**	**Universitätsklinikum Würzburg, Department of General Practice**
**Date**	**15-Sep-2024**
**COI**	**None**

Dear Editor, Dear Authors

Thank you for allowing me to review this interesting manuscript, dealing with an important topic in primary care research, i.e. beliefs of the lay public of duration of common illness. Gaining methodologically valid insides on these beliefs is key to understand and address patients' expectations during routine consultations, therefore, to provide better patient-centered care. The manuscript is clearly written, descriptive statistics although simple are appropriately used, and visualisation is very good and readily understandable.

I recommend accepting this good quality manuscript. Besides some mistyping (e.g. P. 6 line 13 "… t they…" and some others), at the editor's and authors' discretion, the manuscript could be improved following some minor (optional) suggestions:

1) Abstract – conclusions: name the infections, e.g. in brackets: "Participants underestimated the duration of some infections compared (which?) to evidence-based estimates and were accurate in their estimates for other infections (which?).";

"Many stated that they would not be…": how many? For which infections?

2) Strengths and limitations box could be improved in the wording, e.g. a) bullet point 1 and 4 merged in a coherent sentence (I agree this is a conveniance sample, although with

representative quotas), b) elaborate a bit further why bullet point three is a limitation and bullet point two is a strength. Instead of pointing out that it was the first Australian survey, a “stronger strength” would be, maybe, that it is one of very few, it captures beliefs during/after COVID (as not previously done), with one limitation being that it is only Australia-based, and being prone to selection bias

3) I would expand the sample size calculation paragraph, as such it is not readily reproducible.

4) On page 6 you mention analysis of free text responses, you can add some precentages in brackets (“the most frequently (?%)…”) to improve clarity and mention these analyses in the methods section. In alternative: you may want to skip this paragraph as it does not add many relevant findings. Otherwise you may want, if data allows, to have a separate paragraph with free text analysis responses that explains a bit more in depth your other interesting quantitative findings.

5) P 6 line 17 what is “this length of time”? unclear

6) P 6 line 24 unclear to me is if the last three sentences refer to the free text analysis responses or how these results were generated? It seems a very “qualitative/interpretative” way of reporting results (e.g. “… were similar to those…”, line 24-25), maybe this kind of sense making reasoning is best placed in the discussion.

7) Strengths and limitation: I would not state that the sample is “representative for” to avoid confusion, a convenience sample cannot be truly representative (a random sample could be), so I would ensure to consistently state that there were representative quotas for age, sex and location.

8) In the discussion you may want to expand on the fact that shared-decision making is a good way to achieve person-centered care (as you pointed out), however, as noted e.g. here (https://www.ncbi.nlm.nih.gov/pmc/articles/PMC7572954/), sometimes this is not realistic due to severe time constraints constraints and reimbursement issues (e.g.), as also recently shown in a qualitative study in UTI (https://bjgpopen.org/content/early/2024/07/08/BJGPO.2023.0224)

**Reviewer**	**2**
**Name**	**Merenstein, Daniel**
**Affiliation**	**Georgetown University**
**Date**	**18-Sep-2024**
**COI**	**NA**

This is a great study. Maybe the best possible study one can see for the methods, ie survey. It is very well written (one minor point below) and again very important, which how often can one say for a survey.

My only question is how evidence based are your references. I didn't look up all the refs and your days look accurate to me but wonder if we can be so confident to sort of imply we have the right answer and are asking patients to guess the right answer. But if large well done MAs then great.

I looked at your flow chart but there was too much jargon to know why so many people were excluded. Seems strange, why not just include as much data as they gave you, even if not complete.

I don't know what to make of the Demographics, ie ½ had a passive approach to healthcare. Ie what is the External Validity for say Americans, Europeans, etc? I don' have the answers, but it is a little concerning.

My only minor issue is feels like the Discussion is too long. You have great point, great findings, not sure it needs so much text. But I defer to the editors.

Overall, what a great study. Simple, well written and to the point.

VERSION 1 - AUTHOR RESPONSE

Reviewer 2		
14	This is a great study. Maybe the best possible study one can see for the methods, ie survey. It is very well written (one minor point below) and again very important, which how often can one say for a survey.	Thank you for your compliments.
15	My only question is how evidence based are your references. I didn't look up all the refs and your days look accurate to me but wonder if we can be so confident to sort of imply we have the right answer and are asking patients to guess the right answer. But if large well done MAs then great.	The evidence comes from the best available evidence for each infection - i.e. systematic reviews and meta-analyses (mainly Cochrane reviews) that provided duration estimates for untreated participants in RCTs as well as systematic reviews that had the primary objective of synthesizing natural history data. These sources of evidence were identified through a scoping review that we conducted (and is published elsewhere). We have provided references to the source of evidence in Figure 1. We acknowledge in the limitations section that while we used the best available evidence for the duration estimates, for some infections (e.g. UTI, impetigo),

		there is limited evidence and some uncertainty in the available evidence.<br><br>Change:<br><br>No Change
16	I looked at your flow chart but there was too much jargon to know why so many people were excluded. Seems strange, why not just include as much data as they gave you, even if not complete.	We have clarified and expanded the reasons for the exclusion. We excluded certain data based on pre-determined quality control measures at the beginning of the study to ensure the integrity of responses and accuracy of the analysis. Despite the numbers excluded, we nonetheless obtained a sample that meets the required sample size for the study.<br><br>**Change**: Supplementary File 3<br><br>1. Participants who failed ReCAPTCHA test (n=51)<br>2. Participants who spent < 8 minutes answering the questions (n=372)<br>3. Participants who failed other quality scores check; straight liners including those who consistently selected the same answers across multiple questions (n=228)<br>4. Disqualified: bad open-ends, profanity (n=48)
17	I don't know what to make of the Demographics, ie ½ had a passive approach to healthcare. Ie what is the External Validity for say Americans, Europeans, etc? I don' have the answers, but it is a little concerning.	We have added to the results and the discussions to clarify this.<br><br>**Change**: Page 5, Lines 176-178<br><br>Just over half (n=346, 59%) indicated, on the Maximiser-Minimiser scale, a preference for a more passive approach to healthcare unless it is necessary.<br><br>Our participants' preference for a less aggressive approach to healthcare on the MMS is like peoples' preferences in previous studies [6-8]. This may have contributed to the decision not to usually seek care for common infections.
18	My only minor issue is feels like the Discussion is too long. You have great point, great findings, not sure it needs so much text. But I defer to the editors.	Thank you for your feedback. We feel that the discussion length is appropriate and within the typical word count of a discussion.
19	Overall, what a great study. Simple, well written and to the point.	Thank you.

References

1. van Duijn HJ, Kuyvenhoven MM, Schellevis FG, Verheij TJ. Illness behaviour and antibiotic prescription in patients with respiratory tract symptoms. *British Journal of General Practice*. Jul 2007;57(540):561-8.
2. Caverly TJ, Hayward RA. Dealing with the Lack of Time for Detailed Shared Decision-making in Primary Care: Everyday Shared Decision-making. *Journal of General Internal Medicine*. Oct 2020;35(10):3045-3049. doi:10.1007/s11606-020-06043-2
3. Kurotschka PK, Hemkeppler J, Gierszewski D, Ghirotto L, Gágyor I. General practitioners' decision making managing uncomplicated urinary tract infections in women: a qualitative study. *BJGP Open*. 2024:BJGPO.2023.0224. doi:10.3399/bjgpo.2023.0224
4. Del Mar C, Hoffmann T, Bakhit M. How can general practitioners reduce antibiotic prescribing in collaboration with their patients? *Australian Journal of General Practice*. Jan-Feb 2022;51(1-2):25-30. doi:10.31128/ajgp-07-21-6084
5. Légaré F, Thompson-Leduc P. Twelve myths about shared decision making. *Patient Education and Counseling*. 2014;96(3):281-286. doi:10.1016/j.pec.2014.06.014
6. Abukmail E, Bakhit M, Hoffmann TC. Exploring individuals' perceptions and acceptability of a 'wait and see' approach for managing self-limiting illnesses: A qualitative study. *Patient Education and Counseling*. Jan 2024;118:108032. doi:10.1016/j.pec.2023.108032
7. Chiu AS, Hoxha I, Jensen CB, Saucke MC, Pitt SC. Medical Maximizing Preferences and Beliefs About Cancer Among US Adults. *JAMA Netw Open*. Jun 3 2024;7(6):e2417098. doi:10.1001/jamanetworkopen.2024.17098
8. Scherer LD, Shaffer VA, Caverly T, DeWitt J, Zikmund-Fisher BJ. Medical Maximizing-Minimizing Predicts Patient Preferences for High- and Low-Benefit Care. *Medical Decision Making*. Jan 2020;40(1):72-80. doi:10.1177/0272989x19891181
